# A Novel M6A-Related Genes Signature Can Impact the Immune Status and Predict the Prognosis and Drug Sensitivity of Lung Adenocarcinoma

**DOI:** 10.3389/fimmu.2022.923533

**Published:** 2022-07-04

**Authors:** Xuewen Wang, Chengfei Zhao, Dandan Huang, Zhoujie Liu, Mengmeng Liu, Fei Lin, Yingyu Lu, Jing Jia, Liqing Lin, Xinhua Lin, Huangyuan Li, Zhiwei Chen

**Affiliations:** ^1^ Department of Preventive Medicine, School of Public Health, Fujian Medical University, Fuzhou, China; ^2^ Department of Pharmacy, School of Pharmacy and Medical Technology, Putian University, Putian, China; ^3^ Department of Pharmaceutical Analysis, School of Pharmacy, Fujian Medical University, Fuzhou, China; ^4^ Department of Pharmacy, First Affiliated Hospital of Fujian Medical University, Fuzhou, China; ^5^ Key Laboratory of Nanomedical Technology (Education Department of Fujian Province), School of Pharmacy, Nano Medical Technology Research Institute, Fujian Medical University, Fuzhou, China; ^6^ Fujian Provincial Key Laboratory of Environment Factors and Cancer, School of Public Health, Fujian Medical University, Fuzhou, China; ^7^ Fuzhou Center for Disease Control and Prevention, Fuzhou, China

**Keywords:** immune microenvironment, prognosis, lung adenocarcinoma, m6A related genes, nomogram

## Abstract

Lung adenocarcinoma (LUAD) is a primary cause of cancer-related death around the world and has a poor outcome and high incidence. Treatment options are, however, restricted. One of the most critical factors in cancer and metastasis is the N6-methyladenine (m6A) alteration on RNA. This modification could alter gene expression and even function at numerous levels, such as the stability, translocation and translation of RNA splicing. This study aimed to construct an m6A-related genes signature to accurately predict the prognosis of LUAD patients. From TCGA datasets, the LUAD patient data and m6A-related genes were retrieved. LUAD patients’ mutational features and differentially expressed genes (DEGs) were investigated. An univariate and LASSO model with m6A-related genes were constructed for the prediction of outcomes in LUAD. It was possible to develop a prognostic nomogram that could quantitatively predict LUAD patients’ overall survival chances at 1, 3, and 5 years. Research into biological processes and cell pathways was carried out using GSEA. This study found six m6A-related DEGs in LUAD patients, and three of these DEGs(HNRNPC, IGFBP3 and IGF2BP1) were linked to the clinical outcomes of LUAD patients. We found that the overall survival rate for all LUAD patients with high-risk subgroup was considerably lower. According to ROC curves, the prognostic signature demonstrated a high degree of accuracy in predicting future outcomes. In addition, we created a novel nomogram achieved great accuracy with this one as well. The researchers also found that the novel signature might favorably modulate the immune response, and high-risk scores samples were more susceptible to numerous chemotherapeutic medicines. Overall, we developed a m6A-related gene prognostic signature that effectively predicted outcomes of LUAD patients and gave an immunological perspective for creating customized therapeutics.

## Introduction

1.76 million people die from lung cancer each year, making it the most common cause of death in the world (1). Worse still, lung cancer’s incidence and death are both increasing (2). Lung adenocarcinoma (LUAD), which accounts for almost half of all kinds of lung cancer based on histology and prognosis, is on the rise, particularly in women and young adults (3). Overall LUAD survival remains dismal in spite of considerable advances in treatment modalities including surgical treatment, targeted therapy and early cancer identification (4, 5). LUAD cannot be detected early by current cytology and imaging screenings, despite their high sensitivity as cancer screening methods (6). Therefore, identifying reliable biomarkers for the prediction of the outcomes of LUAD patients is an absolute necessity.

More than 160 types of post-transcriptional chemical changes have been discovered in diverse RNAs, according to the 2017 MODOMICS report (7). N6-methyladenosine (m6A), firstly discovered in the 1970s, is the most prevalent and abundant posttranscriptional alteration found in eukaryotic mRNA, according to this research (8). Every component of the RNA metabolism is thought to be affected by M6A methylation (9, 10). Three types of enzymes control M6A modifications: “writers” (methyltransferases such as METTL3/14/16, RBM15/15B, KIAA1429 and WTAP), “readers” (YTH domain containing RNA binding proteins and heterogeneous nuclear ribonucleoproteins such as HNRNPA2B1, HNRNPC, YTHDC1 and YTHDF1/2/3) and “erasers”. (demethylases, including FTO and ALKBH5) (11–13). M6A has been linked to a wide range of malignancies, and it was believed to be a key player in tumor development and progression (14–16). M6A-related genes’ potential as new biomarkers has also piqued the interest of researchers.

In this study, we aimed to construct an m6A-related genes signature to accurately predict the prognosis of LUAD patients. Our group used bioinformatics and statistics to create a m6A-related gene prognostic signature based on data from TCGA database to reliably predict the outcomes of LUAD patients. An m6A-associated gene-based prognostic signature was discovered to have a high level of predictive power. Furthermore, a nomogram was developed to objectively predict the overall survival (OS) of LUAD patients.

## Materials and Methods

### Chip Data

RNA‐seq mRNA expression profiles and clinical information of TCGA‐LUAD cohorts were downloaded from TCGA platform. Pairs of normal samples were initially extracted from TCGA-LUAD cohorts using their barcodes. All datasets included consisted of 535 LUAD samples and 59 adjacent non-cancerous samples. Then, FPKM values were converted into transcripts per million (TPM) values (TPM). Analysis of numerous samples from the same patients yielded an average expression value. **Supplementary Table 1** displays the clinical data of all LUAD patients. From the literature and from the m6Avar database, M6A-related genes that were linked to LUAD were collected (http://m6avar.renlab.org/) (**Supplementary Table 2**).

### Cell Lines and Cell Transfection

All cell lines (16-HBE, NCI-H1299, NCI-H1703, NCI-H2126, NCI-H460, SPC-A1 and A549) were obtained from the Chinese Academy of Sciences (Shanghai, China). Cells were grown in RPMI 1640 nutrient solution (Gibco, USA). There was 10% FBS in all the media. All cell lines were grown in 5% CO_2_ at 37°C for the duration of the study.

ComiFECT transfection reagent was used for the cell transfection (Comiike, Nantong, Jiangsu, China). Silent IGF2BP1-targeting siRNAs (si-NC) and negative controls were bought from Genomeditech Co., Inc.

### RT‐PCR

TRIzol^®^ reagent (Invitrogen, Shanghai, China) was applied to extract the total RNA from LUAD cells, and 300 ng extracted RNAs were reverse transcribed into cDNA by the use of ReverTra Ace qPCR RT Kit (Toyobo, China). THUNDERBIRD SYBR^®^ qPCR Mix (Toyobo, Japan) was used for quantitative PCR (Roche, Shanghai, China). The GAPDH was applied as an endogenous control mRNA for normalizing the expressions of targeting mRNAs. Each sample was examined three times. Data from curves was then gathered to confirm the specificity of the PCR. The relative expression fold change of miRNAs was calculated by the 2^-ΔΔCt^ methods. Primer sequences were as follows: IGF2BP1, 5’-GCGGCCAGTTCTTGGTCAA-3’ and 5’- TTGGGCACCGAATGTTCAATC-3’; GAPDH, 5’- ACAACTTTGGTATCGTGGAAGG -3’ and 5’- GCCATCACGCCACAGTTTC -3’.

### Cell Counting Kit-8 (CCK-8) Assay

Cell viabilities were examined by the use of the CCK-8 kit (FineTest, Wuhan Fine Biotech Co., Ltd, Wuhan, Hubei, China). After the transfections, 100 µL cells (5×10^3^ cells per well) were seeded in 96-well plates. At 0, 24, 48, and 72 hours, 10µL of CCK-8 solution was added to each well. A microplate reader was applied to examine the absorbance at 450 nm after 1 hour of incubation.

### Transwell Assay

NCI-H460 and NCI-H1299 cells transfected with si-IGF2BP1 and its corresponding control cells were seeded onto pre-treated Matrigel. 500 μL and 100 μl of culture medium were added into the upper and lower chambers, respectively. 24 h later, the cells were stained with 0.1% crystal violet. Subsequently, a microscope was applied to observe cell staining.

### Extraction of M6A-Related Gene Matrix and Identification of Differentially Expressed Genes (DEGs)

The expressing matrix of TCGA genes was selected to extract M6A-related genes expression patterns. The DEGs were discovered through the use of the R program ‘limma’, with the log2 fold-change (log2 FC) criterion of more than 1.5 and the false discovery rate (FDR) less than 0.05.

### Selection of Potential Survival-Associated Genes

With the help of the survival packages, we ran a univariate cox analysis on all of the DEGs. In accordance with this classification, DEGs with p-values less than 0.05 were designated prognostic-associated genes and identified as candidate genes for further investigation as a result of the classification process.

### Developments of a Prognostic Model

We employed LASSO to create a better risk score model in order to better forecast m6A genes and LUAD. In the next step, we used R’s survival and glmnet packages to perform LASSO assays on TCGA’s candidate genes. Finally, the genes and their coefficients were figured out. On the basis of the established prognostic model, LUAD patients were categorized into high-risk (median) and low-risk (median) groups. The OS differences were compared using Kaplan-Meier assays and the log-rank tests. The “survivalROC” packages were used to produce the time-dependent ROC curve, which was then used to test the predicted accuracy of the prognostic risk score mode of operation (17).

### Cluster Analysis and Principal Component Analysis

The cluster analysis was used to construct a principal component analysis (PCA). In addition, clinical data were retrieved from LUAD specimens for further analysis. In the following step, the R software was used to conduct a correlation analysis between clinical features and clustering results. Once everything was finished up, the heatmap was constructed using the R computer language’s ggplots package.

### GSVA

R package “GSVA” was used to run GSVA on the gene profile in order to compare the differences in biological processes between low- and high-risk groupings of the risk score (18). It was possible to utilize the GSVA approach, which is non-parametric and unsupervised, to evaluate pathway changes or biological processes when an expression matrix sample was provided as input. “c2.cp.kegg v7.1 symbols” gene sets were utilized as the reference gene sets in this study.

### Developments of a Novel Nomogram

The “rms” package in R was used to create a nomogram that included age, gender, pathological stage, and a predictive risk score model based on the TCGA cohort. The nomogram’s accuracy was predicted. To test whether the model could be utilized as an independent indicator for predicting LUAD in LUAD, multivariate Cox regression was performed. Following the online ROC curves, the nomogram’s AUC was determined to indicate the nomogram’s prognostic value.

### Tumor-Infiltrating Immune Cells (TICs) Profile

On 535 tumor and non-tumor samples, the CIBERSORT method was used to compute the relative amounts of 22 TICs in each LUAD sample; samples with P 0.05 were used for further investigation (19).

### Evaluation of Drug Sensitivity

The 50% inhibitory concentration was known as the IC50. An R program called “pRRophetic” was used to determine the IC50 of 138 medications by using its dependencies such as “car,” “ridge preprocessCore,” “genefilter, and sva.” (20). The “ggplot2” R package was used to generate the boxplot.

### Functional Enrichment Analysis

ClusterProfiler, a R program, was used to perform pathway enrichment analyses for patients in the high- and low-risk groups using Gene Ontology (GO) and the Kyoto Encyclopedia of Genes and Genomes (KEGG) (21). In terms of statistical significance, GO keywords and KEGG pathways with P values less than 0.05 were found.

### Statistical Analysis

R (version 3.6.1; R Foundation for Statistical Computing, Vienna, Austria) software was used to conduct statistical analysis. For the data matrix and all of the data processing, Perl was utilized. The “limma” R package was used to identify m6A-related genes that differed in expression. R packages “survival” and “survminer” were used to perform Cox regression and survival analysis. Using Kaplan-Meier analysis, we looked at the variations in survival rates between the two risk categories. LUAD’s OS was predicted using an independent set of indicators found through a Cox regression study. The prognostic risk score mode and nomogram were tested for their predictive power using ROC curves. P 0.05 with a two-tailed test was deemed significant.

## Results

### Identification of the m6A-Related DEGs in LUAD

**Figure 1:**
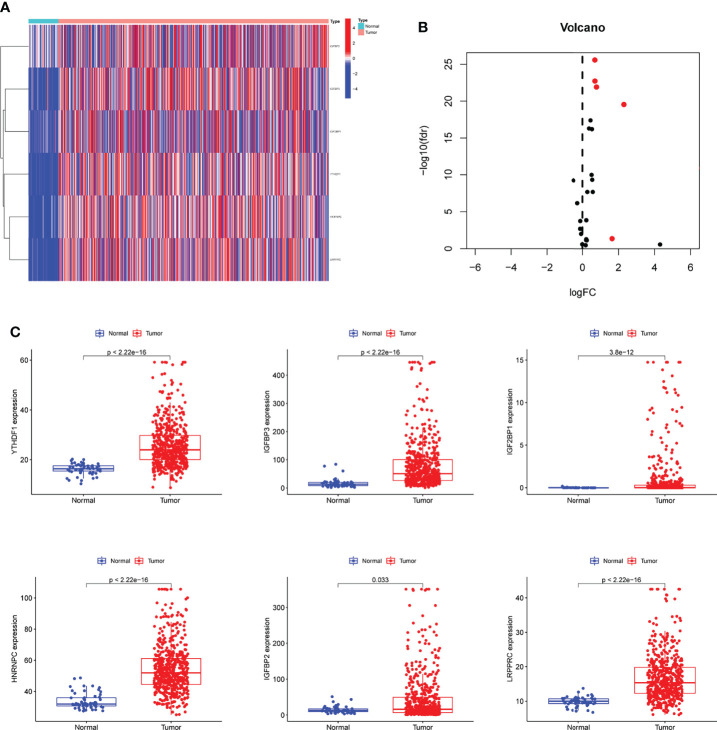
The identification of m6A-related DEGs in LUAD. **(A, B)** Heat Map and Volcanic map of m6A-related DEGs between LUAD and control samples with log2 FC>1.5 and p<0.05. **(C)** All 6 m6A-related DEGs were distinctly increased in LUAD specimens compared with non-tumor specimens.

Firstly, we downloaded the names of m6A-related genes, and then performed limma using TCGA datasets. The results showed that six m6A-related DEGs were identified between LUAD specimens and non-tumor specimens (**Figures 1A, B**). In addition, all seven genes were distinctly increased in LUAD specimens compared with non-tumor specimens, including IGFBP2, IGFBP3, IGF2BP1, YTHDF1, HNRNPC and LRPPRC (**Figure 1C**). Our findings suggested that the seven genes may be functional regulator in progression of LUAD.

### Construction and Evaluation of an m6A-Related Genes Prognostic Signature

**Figure 2:**
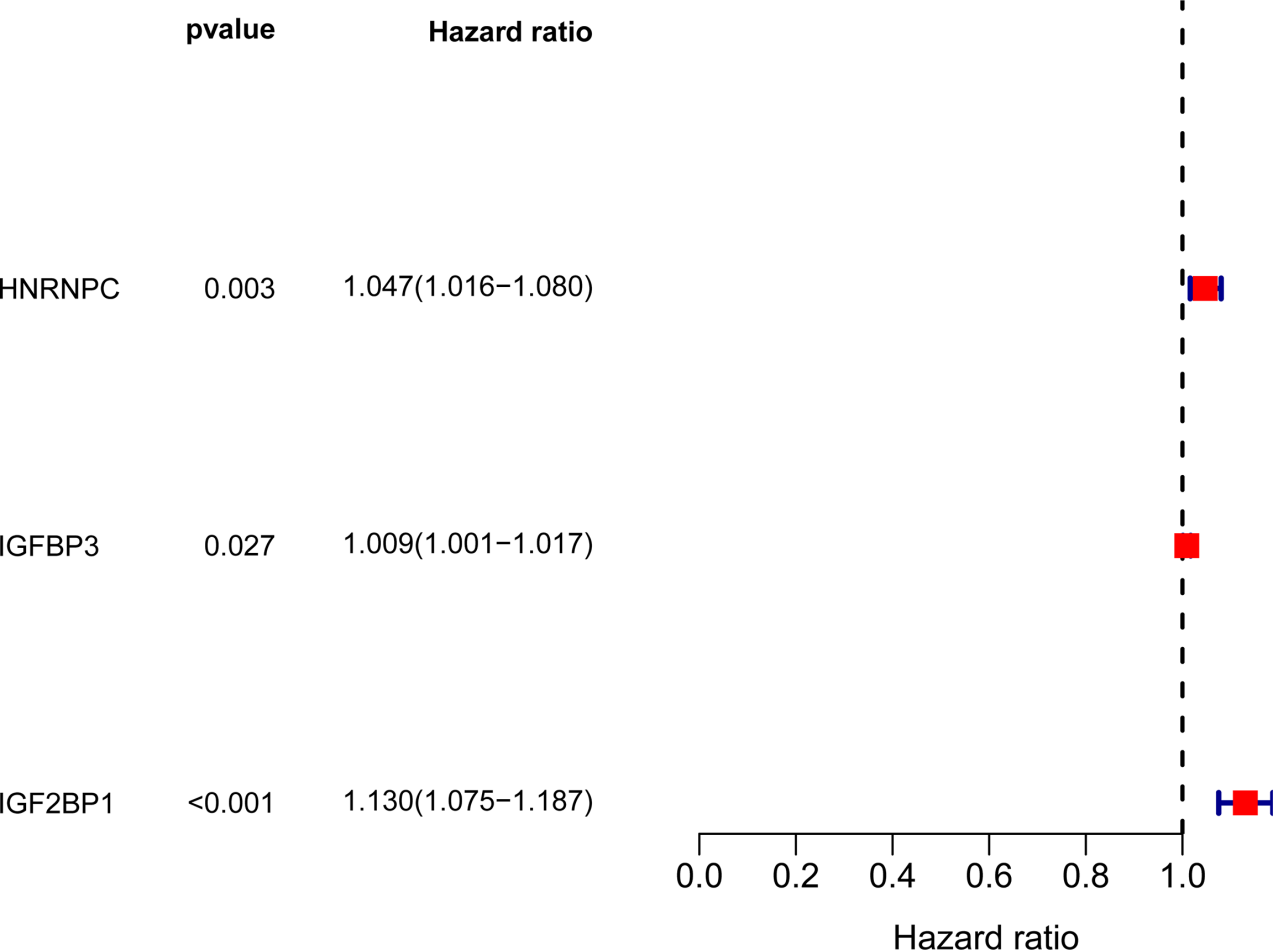
Forrest plot of 3 m6A-related DEGs related with prognosis by univariate Cox proportional hazards regression analysis.

**Figure 3:**
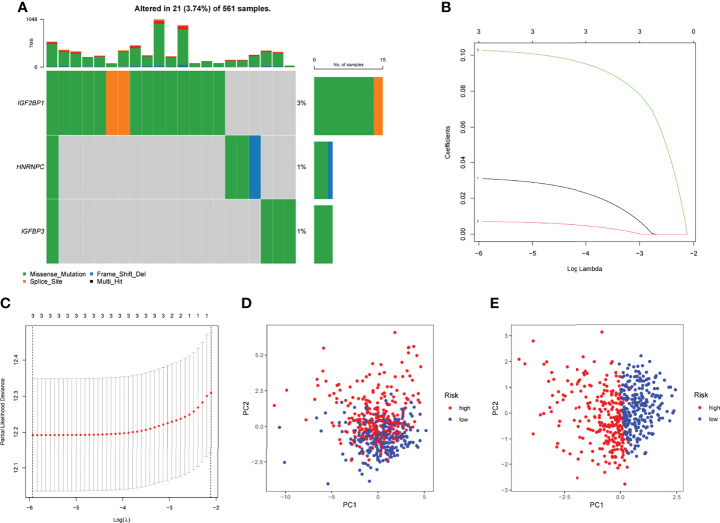
Developments of prognostic model. **(A)** The mutation frequency of 3 m6A-related DEGs in LUAD patients from TCGA datasets. **(B)** LASSO coefficients of the 3 m6A-related DEGs. **(C)** Identifying genes for the creation of a model for predicting prognosis. **(D)** Principal component assays using m6A-related DEGs in LUAD. **(E)** Tumors and normal samples in the TCGA cohort can be distinguished using principal component analysis.

In TCGA datasets, we performed univariate assays using the six m6A-related DEGs to develop a prognostic signature for LUAD patients. DEG expression was found to be substantially linked with LUAD patient outcome (**Figure 2**). An overview of three m6A-related DEGs associated with poor prognosis was provided. The somatic mutation profile m6A-related gene alterations were found in 21 out of 561 LUAD samples, or a frequency of 3.74 percent, as shown in **Figure 3A**. For the sake of avoiding overfitting, LASSO assays were applied to exclude these strongly linked predictive DEGs, and three m6A-related genes were discovered. (**Figures 3B, C**). The risk score of each sample was calculated by the use of the following: risk score = (0.0310199095911482) ×HNRNPC+(0.00708641474163214) ×IGFBP3 +(0.102677930685888) ×IGF2BP1. In order to separate LUAD samples completely, the risk score model was employed (low or high risk) (**Figures 3D, E**).

### The Prognostic Value of Novel Risk Model in LUAD Patients

**Figure 4:**
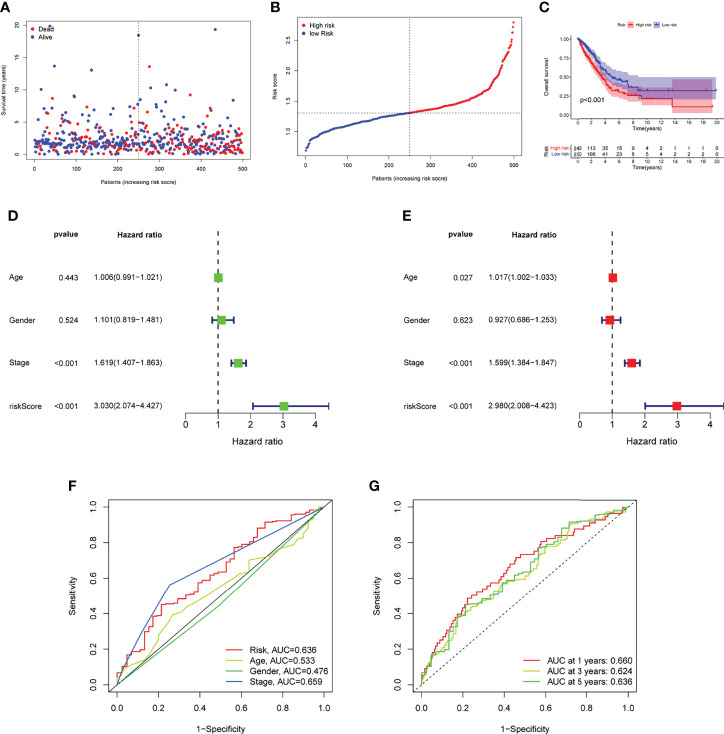
ROC analysis, risk score analysis, and survival analysis for LUAD’s three-gene signature are discussed. **(A)** Patients’ long-term survival rates in low- and high-risk groups **(B)** Distributions of risk scores. **(C)** Based on the entire TCGA cohort, the Kaplan-Meier curves of OS between low-risk and high-risk groups **(D**, **E)** Univariate and multivariate assays for the signature established by TCGA datasets. **(F)** ROC assays for different clinical factors and risk score. **(G)** Test results showed that the signature performed as expected in TCGA datasets.

**Figure 5:**
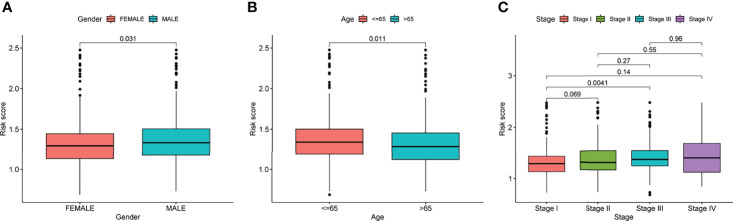
The relationships of risk score and clinical factors, including **(A)** gender, **(B)** age, and **(C)** Stage.

Prognostic risk-related signatures for LUAD patients were classified to low- and high-risk groups based on the median value of their risk scores in TCGA datasets (**Figures 4A, B**). Patients in the high-risk group had a considerably lower overall survival rate than those in the low-risk group, according to survival tests (**Figure 4C**). The risk score and stage of LUAD patients were found to be strongly linked with their OS in a univariate study (**Figure 4D**). More interestingly, both risk score and clinical stage were independent predictors of OS in multivariate assays, whereas risk score and stage were only linked with OS in the univariate study (**Figure 4E**). The overall predictive power of the risk model for overall survival in TCGA datasets was tested using a time-dependent ROC. AUC findings verified the diagnostic usefulness of the tests and we observed that ROC assays may predict a highest accuracy at 1 year (**Figures 4F, G**). In addition, we explored the association between risk score and clinical factors in LUAD patients. We did not observe a distinct difference between risk score and gender and age (**Figures 5A, B**). However, we found that LUAD patients with advanced stages showed a higher value of risk score (**Figure 5C**). Our results revealed that the risk model could be used as a novel prognostic biomarker for LUAD patients.

### A Nomogram Predicting Survivals

**Figure 6:**
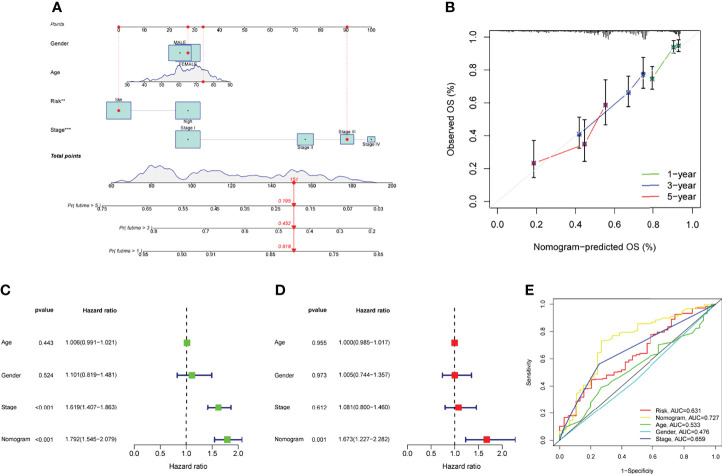
The ability of a risk score and clinical pathological factors to predict the outcome of individuals with LUAD. **(A)** A nomogram that predicts the survival rate of patients with LUAD. **(B)** The calibration plots of the nomogram. **(C)** Univariate Cox regression analysis of the nomogram. **(D)** Multivariate assays of the nomogram. **(E)** ROC curves for clinical pathological features and risk score measurements.

We developed a nomogram for predicting OS in LUAD samples using a predictive risk score model that took into account factors such as gender, age, and clinical stage (**Figure 6A**). The nomogram’s ability to reliably predict the OS of LUAD patients was demonstrated by the calibration curves at one year, three years, and five years, as shown in **Figure 6B**. Multiple Cox regression analyses showed that the prognostic risk score model and the ages as well as the clinical-pathological stages were independent predictors of outcome (**Figures 6C, D**). The nomogram (AUC = 0.727) showed a superior predictive value than a single indicator (**Figure 6E**).

### Assays of the Immune Microenvironment

**Figure 7:**
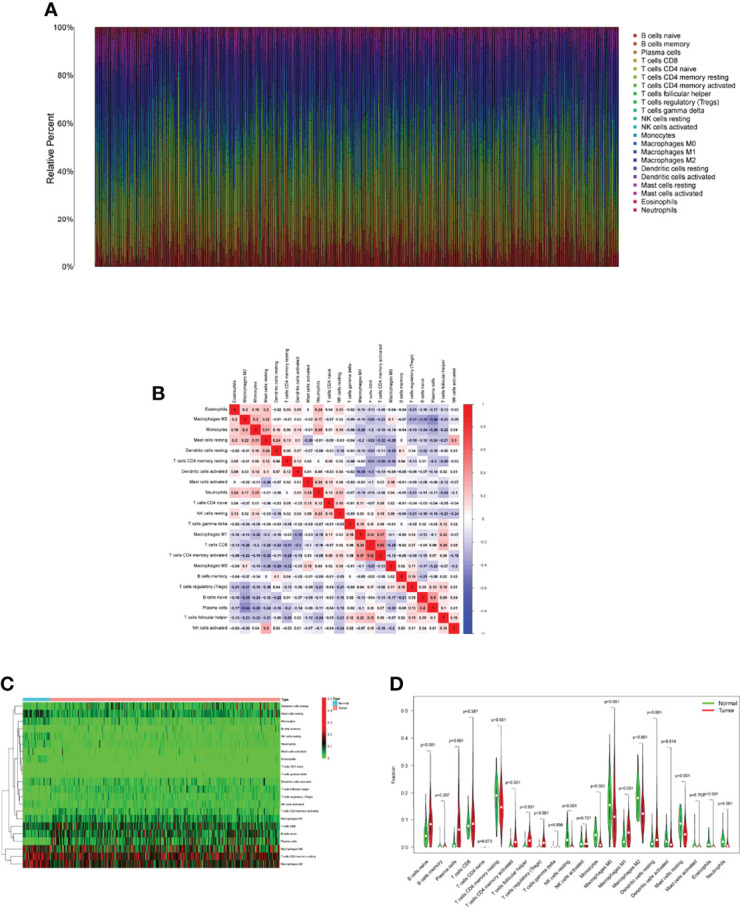
Tumor TICs profiles and association studies. **(A)** The percentage of 22 different types of TICs in LUAD specimens was depicted in a bar graph. **(B)** Barplot showing the proportion of 22 kinds of TICs in LUAD specimens. **(C)** The levels of 22 kinds of TICs in LUAD samples and normal samples. **(D)** Several types of TICs were observed to be increased in LUAD specimens.

**Figure 8:**
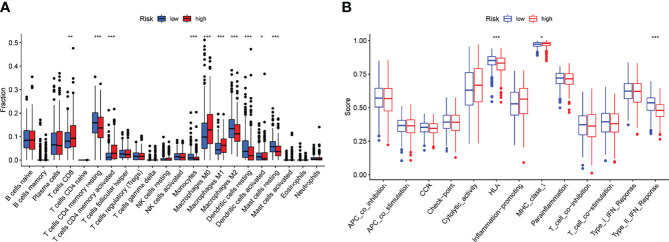
Risk score and fraction of TICs are correlated. **(A)** The variation in immune infiltration between high- and low-risk scores. **(B)** Patients with a high-risk score have a known function in immune regulation that differs from those with a low-risk score. *p<0.05, **p<0.01, ***p<0.001.

Tumor immune cell infiltration is the movement of immune cells into tumor tissue from the circulation (22). Clinical outcomes are strongly linked to the presence of immune cells in tumors, which makes them ideal targets for new cancer treatments (23, 24). Further evidence that the immune microenvironment correlates with risk score was obtained by examining the percentage of tumor-infiltrating immune subsets using the CIBERSORT algorithm and constructing 21 different immune cell profiles in LUAD samples (**Figures 7A, B**). Heat map and Histogram showed the expressing pattern of tumor-infiltrating immune cells in LUAD samples and normal lung samples (**Figures 7C, D**). Patients in the high-risk group had higher ratios of T cells CD8, T cells CD4 memory resting, Monocytes, Macrophages M0 and Macrophages M1 and than those in the low-risk group. However, patients in the low-risk group had higher ratios of T cells CD4 memory activated, Macrophages M2, Dendritic cells resting, Dendritic cells activated and Mast cells resting (**Figure 8A**). Moreover, HLA, Type_II_IFN_Reponse and MHC_class_I were also activated in the low-risk group (**Figure 8B**).

### Response to Chemotherapy Response

**Figure 9:**
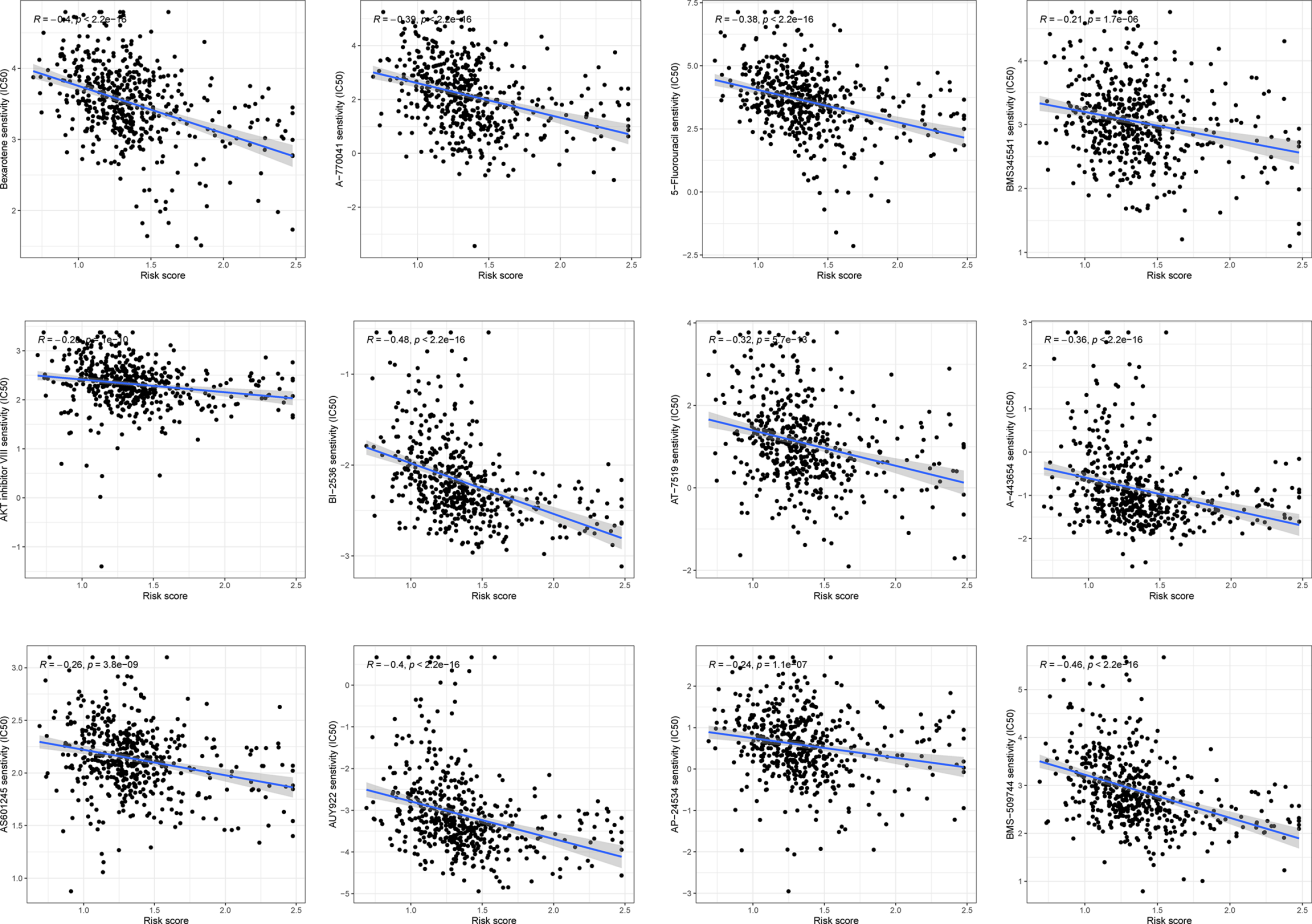
The association between risk score and chemosensitivity.

The correlation between chemoresistance and risk score was investigated since risk score was related to a bad outcome. As shown in **Figure 9** and **Supplementary Table 1**, we discovered that certain chemotherapy medicines had a greater sensitivity to high-risk score samples.

### Gene Set Variation Analysis (GSVA)

**Figure 10:**
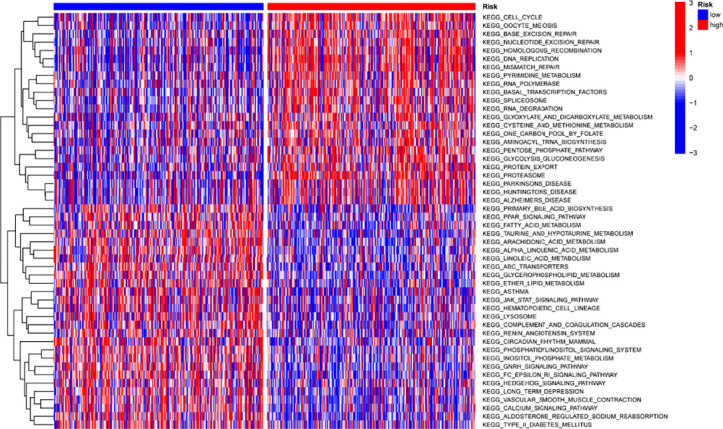
Map of GSVA enrichment between low- and high-risk score categories.

It was done by using “c2.cp.kegg.v7.2” gene sets downloaded from the Molecular Signatures Database (MSigDB) to study the biological behavior of two groups. The high-risk score was found to have a higher concentration of tumor-related pathways (**Figure 10**).

### Functional Correlation Analysis

**Figure 11:**
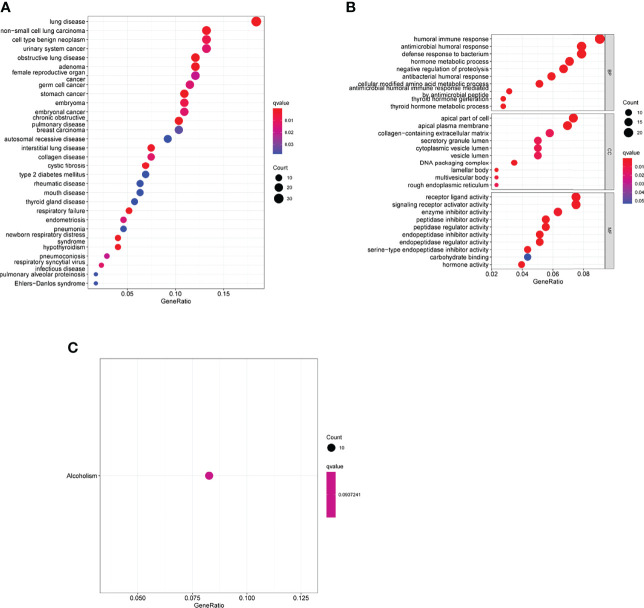
Biological processes were identified by functional enrichment analysis. **(A)** Disease ontology enrichment analysis, **(B)** GO assays and **(C)** KEGG assays of DEGs between high-risk group and low-risk group.

We then compared the expressing patterns of the low and high-risk groups. To understand the function of dysregulated genes, DO pathway enrichment studies were performed. The results indicated that diseases enriched by the dysregulated genes were mainly associated with lung disease, non-small cell lung carcinoma, cell type benign neoplasm, urinary system cancer and obstructive lung disease (**Figure 11A** and **Supplementary Table 3**). GO assays revealed that the dysregulated genes were mainly enriched in humoral immune response, defense response to bacterium, hormone metabolic process, apical part of cell, apical plasma membrane, secretory granule lumen, receptor ligand activity and enzyme inhibitor activity (**Figure 11B** and **Supplementary Table 4**). KEGG assays indicated that the dysregulated genes were mainly enriched in Alcoholism (**Figure 11C**).

### The Oncogenic Roles of IGF2BP1 in LUAD Growth

**Figure 12:**
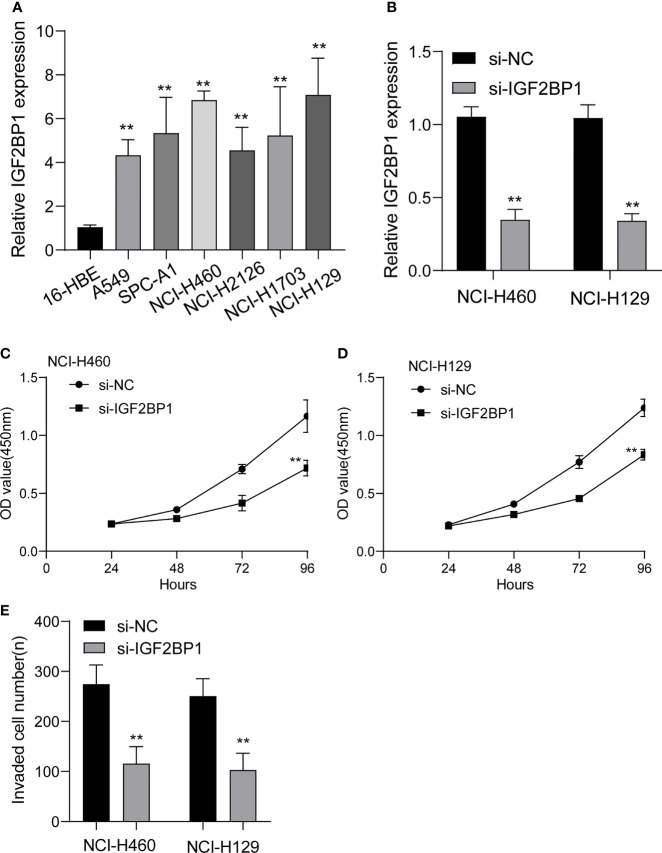
The expressions of IGF2BP1 in LUAD cells and its function. **(A)** RT-PCR for the expression of IGF2BP1 in six LUAD cells and 16-HBE cells. **(B)** qRT-PCR analysis of IGF2BP1 expression after transfection of si-IGF2BP1. **(C, D)** CCK-8 assays were used to explore the function of IGF2BP1 silence on NCI-H460 and NCI-H1299 cells. **(E)** Transwell assays were used to determine the invasive ability of LUAD cells. **p<0.01. The experiments were repeated three times, and each experiment was triplicated.

To study the function of IGF2BP1 in LUAD, we firstly performed RT-PCR to examine its expression in LUAD cell lines. As shown in **Figure 12A**, we found that IGF2BP1 expression was distinctly increased in LUAD cells, including NCI-H1299, NCI-H1703, NCI-H2126, NCI-H460, SPC-A1 and A549, compared with 16-HBE. Moreover, we decreased IGF2BP1 expression in NCI-H460 and NCI-H1299 cells using siRNA, and RT-PCR confirmed the transfection efficiency (**Figure 12B**). In addition, CCK-8 assays confirmed that silence of IGF2BP1 distinctly inhibited the proliferation of NCI-H460 and NCI-H1299 cells (**Figures 12C, D**). Finally, we also observed that knockdown of IGF2BP1 distinctly inhibited the migration of NCI-H460 and NCI-H1299 cells (**Figure 12E**).

## Discussion

Prognostic markers and therapeutic targets have been regularly discovered thanks to advances in high-throughput sequencing technologies over the past few decades (25, 26). As a result, we now know more about cancer. Reliable tumor immunotherapy response and prognostic biomarkers based on the intrinsic milieu of tumorgenesis are still extremely rare in LUAD (27, 28). Research into the mechanisms of action of these compounds is essential for their potential medicinal application. In eukaryotic cells, m6A is by far the most common RNA modification found within the RNA itself (10). RNA methylation in the m6A region appears to have an important role in cancer development, according to newly discovered data (29, 30).

Many M6A-related genes have recently been linked to the progression of various cancers. For instance, colorectal cancer metastatic tissues with increased METTL3 expression were related with a worse prognosis. Through a m6A-IGF2BP2-dependent pathway in colorectal cancer cells, METTL3 knockdown significantly decreased *in vitro* cell self-renewal, frequency of the stem cell population, and migration, as well as colorectal carcinoma tumorigenesis and metastasis (31). Chen et al. reported that hepatocellular carcinoma patients with high WTAP expression displayed a worse outcome, and WTAP expression could be an independent predictor of survival. WTAP increased hepatocellular carcinoma cell proliferation and tumor growth *in vitro* and *in vivo via* the m6A-HuR-dependent epigenetic silencing of ETS1 *in vitro* and *in vivo (* 32). Importantly, Wang and his group reported that increasing the cisplatin response by overexpressing IGFBP3 promoted apoptosis and confirmed that suppression is caused in part by inhibiting IGF1 signaling *in vitro (* 33). These findings indicated the critical roles of M6A-related genes in the progression of various cancers. In this study, we analyzed TCGA datasets and identified six dysregulated M6A-related genes in LUAD. The results of Univariate indicated that only three M6A-related genes were survival-related genes, including HNRNPC, IGFBP3 and IGF2BP1. HNRNPC, IGFBP3, and IGF2BP1 were created as a three-gene prognostic signature that performed well in predicting the survivals of patients. With the addition of a few selected clinical and pathological parameters, the predictive power of this prognostic risk score model was significantly enhanced. Then, we chose IGF2BP1 to study its potential function. The results indicated that diseases enriched by the genes involved in the expression of GF2BP1 were mainly associated with lung disease, non-small cell lung carcinoma, cell type benign neoplasm, urinary system cancer and obstructive lung disease, suggesting that GF2BP1 may play an important role in the progression of LUAD. Then, functional assays revealed that IGF2BP1 knockdown suppressed the proliferation and invasion of LUAD cells, which may explain the reason that IGF2BP1 was associated with poor prognosis of LUAD patients.

To better understand carcinogenesis and cancer progression, researchers are increasingly focused on the tumor environment (TME), which has risen to prominence as a research hotspot in recent years (34, 35). In addition, emerging data suggests that tumor-infiltrating immune cells (TICs) and stromal components are strongly linked to the development of LUAD (36, 37). Carcinogenesis and development of cancer were greatly influenced by the tumor microenvironment, particularly the immunological component. It has been found that shifting the TME from a tumor-friendly to a tumor-suppressive state can benefit cancer treatment (38, 39). As a result, identifying the prospective therapeutic targets that contribute to the aforementioned process is an absolute necessity. In this study, we observed that high-risk score patients were enriched with inhibitory immunity cells. HLA and MHC class I activation, as well as inflammatory-promoting activity, were seen in patients with a high-risk score, indicating that individuals with a high-risk score can benefit from immunotherapy. On the other hand, to better understand the relevance of the predictive risk score model in LUAD, the variations in patients’ responses to pharmacological therapy between low- and high-risk groups were studied. According to the preceding definitions, patients with high-risk scores showed a considerable stroma activation status, indicating chemoresistance.

Several issues remained in the current study. First, the number of patients was quite small. Second, the prognostic model has to be tested on a large number of different datasets in order to ensure its robustness. Third, some possible risk variables, including radiation and pathological characteristics, were not included in our nomogram. Finally, these prognostic M6A-related genes in LUAD need additional investigation to understand their function and processes.

## Conclusion

A predictive signature based on three M6A-related genes was created to predict the overall survival of LUAD patients. Our developed signature of three M6A-related genes gives higher clinical utility for predicting the prognosis of LUAD patients compared to the usual TNM staging approach. Our findings will lead to the developments of individualized cancer chemotherapy and immunotherapy in the future.

## Data Availability Statement

The datasets presented in this study can be found in online repositories. The names of the repository/repositories and accession number(s) can be found below: https://portal.gdc.cancer.gov/, TCGA-LUAD.

## Author Contributions

All authors made a significant contribution to the work reported, whether that is in the conception, study design, execution, acquisition of data, analysis and interpretation, or in all these areas; took part in drafting, revising or critically reviewing the article; gave final approval of the version to be published; have agreed on the journal to which the article has been submitted; and agree to be accountable for all aspects of the work.

## Funding

The authors gratefully acknowledge the financial supports of the National Natural Science Foundation of China (81973083, 22074017 and 81703477), Natural Science Foundation of Fujian province (21J02034).

## Conflict of Interest

The authors declare that the research was conducted in the absence of any commercial or financial relationships that could be construed as a potential conflict of interest.

## Publisher’s Note

All claims expressed in this article are solely those of the authors and do not necessarily represent those of their affiliated organizations, or those of the publisher, the editors and the reviewers. Any product that may be evaluated in this article, or claim that may be made by its manufacturer, is not guaranteed or endorsed by the publisher.

## Supplementary Material

Supplementary Figure 1 The association between risk score and chemosensitivity.

Supplementary Table 1 The clinical data of all LUAD patients from TCGA datasets.

Supplementary Table 2 M6A-related genes.

Supplementary Table 3 DO pathway enrichment.

Supplementary Table 4 GO pathway enrichment.
