# Supplementary figures and images for: A Novel M6A-Related Genes Signature Can Impact the Immune Status and Predict the Prognosis and Drug Sensitivity of Lung Adenocarcinoma
Source: Front Immunol. 2022 Jul 4;13:923533. doi: 10.3389/fimmu.2022.923533 (PMC9289247; doi:10.3389/fimmu.2022.923533)

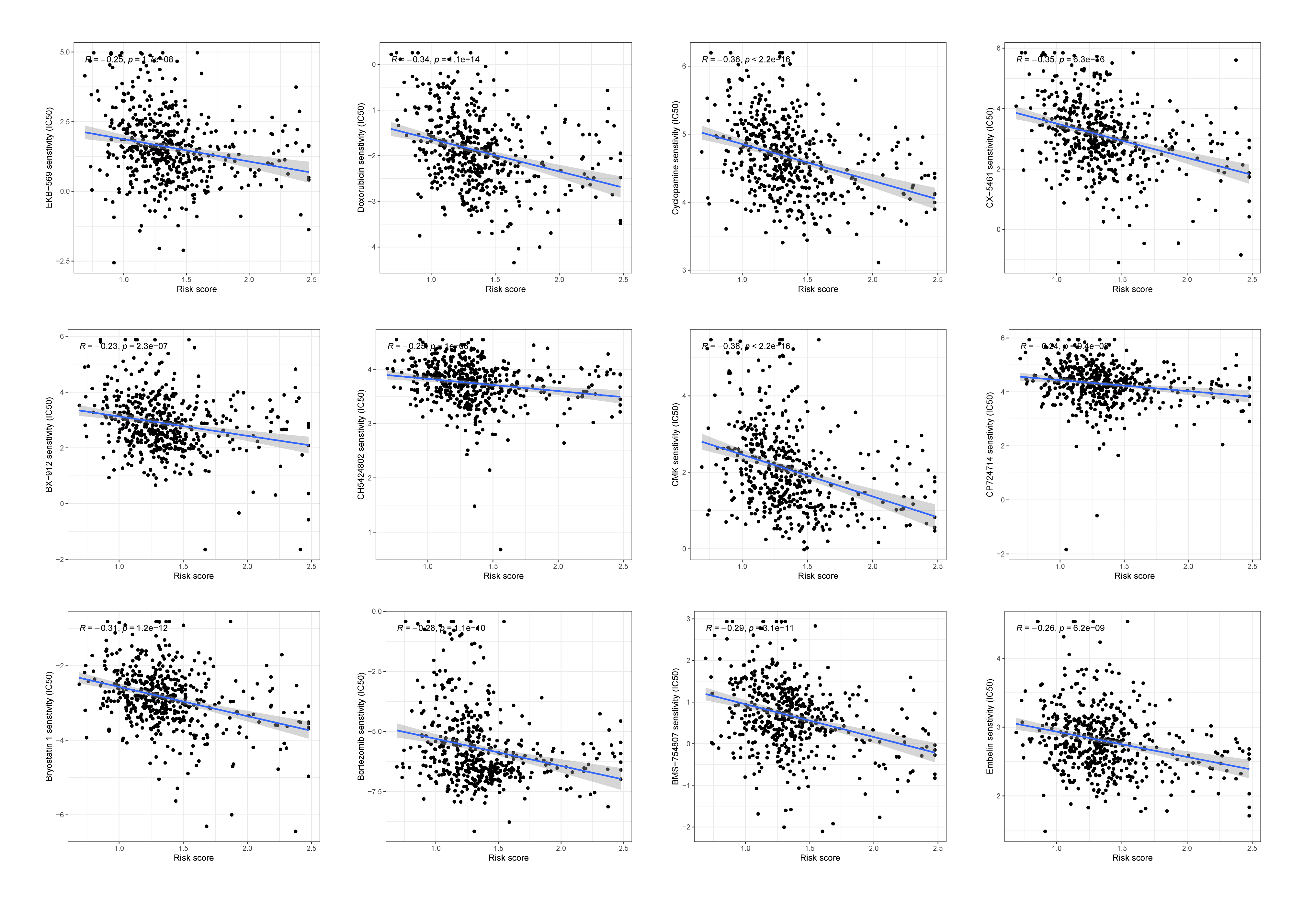

Supplement: Supplementary Figure 1 — The association between risk score and chemosensitivity. [file Image_1.tif]
